# International cooperation to fight cancer’s late-stage presentation in low- and middle-income countries

**DOI:** 10.1007/s10585-022-10196-1

**Published:** 2023-01-17

**Authors:** Oliver Henke, Abdul Qadir Qader, Goodluck Lincoln Malle, Jules Roger Kuiate, Lisa Hennig, Tamiru Demeke, Clara Stroetmann, Antje Anneliese Henke, Tamuedjoun Talom Alaric, Marine Rushanyan, Cornell Enssle, Hermann Bussmann

**Affiliations:** 1 Section Global Health, University Hospital Bonn, Bonn, Germany; 2 Radiology Department, Herat University, Herat, Afghanistan; 3 Marangu Lutheran Hospital, Marangu, Tanzania; 4 Department of Biochemistry, University of Dschang, Foréké-Dschang, Cameroon; 5 Heidelberg Institute of Global Health, University Hospital Heidelberg, Heidelberg, Germany; 6 Addis Ababa University, Addis Ababa, Ethiopia; 7 Institute of Epidemiology, Biometry and Informatics, Martin Luther University Halle-Wittenberg, Halle (Saale), Germany; 8 Evangelical University of Cameroon, Bandjoun, Cameroon; 9 Hematology Center After Prof. R. Yeolyan, Yerevan, Armenia; 10 Windhoek Central Hospital, Windhoek, Namibia; 11 Department of Applied Tumor Biology, University Hospital Heidelberg, Heidelberg, Germany

**Keywords:** Global oncology, Cancer, LMIC, Cooperation, Metastasis

## Abstract

Cancer is becoming a massive public health burden in low- and middle-income countries (LMIC). 70% of all cancer deaths globally are attributed to LMIC while the incidence proportion is below 60%. The main reason for the higher mortality rate is “late-stage presentation” of patients with stage III or IV diseases when being diagnosed. Main reasons for this are limited (financial) resources, poor knowledge of health service provider about cancer, misbelieves and fear among patients as well as low health literacy rate. During the 1st International Conference on Hospital Partnerships, conducted by the German Agency for International Cooperation (GIZ), cancer specialists from seven LMIC and Germany discussed opportunities, challenges and solutions of the development of cancer services. Two days of in-depths discussion identified five topics to be playing a key role in the effort to reduce the cancer burden in LMIC: Health Policy & Financing, Barriers to Access, Capacity Building, Cancer Registries and Adapted Treatment Guidelines. By using mind-mapping technique, stakeholders, core topics, main and important topics were visualized and interconnections displayed. Many topics can be addressed through international cooperations but political willingness and commitment in the respective countries plays the crucial role. An essential contribution will be to assist policy makers in formulating and endorsing affordable and effective health policies. Another lesson learned from this workshop is the similarity of challenges among the participating representatives from different LMIC. The authors of this letter emphasize on the importance of building international long-term cooperations to advance oncology care on a global scale.

**Dear Editors,**

Cancer is already a massive public health burden in low- and middle-income countries (LMIC) and will continue to do so in the future. WHO projections estimate that the cancer incidence in the least developed countries will almost double by the year 2040 [1]. As of now 70% of the global cancer mortality with approximately 7 million deaths occur in LMIC, while less than 60% of the global incidence occurs in these countries mostly located in the global south. This means that people living in the global south have a higher likelihood to die from cancer. Indeed, women in East Africa are among the people having worldwide the highest risk to die from cancer diseases [2].

The reasons are manifold including limited financial resources both on patients and service provider side, poor knowledge about cancer also on the provider side resulting in misdiagnosis, and a generally low health literacy rate among the general population facilitating misbeliefs and trust in alternative and less effective medical practices [3].

Thus “late-stage presentation” is common among cancer patients. The occurrence of locally advanced or even metastasized disease among newly diagnosed cancer cases range from 40% in some Latin American countries to 80% in many African countries [4].

Many international organizations work towards a mitigation of these challenges, among them the German Agency for International Cooperation (GIZ; Gesellschaft für Internationale Zusammenarbeit), a federal enterprise working closely with the German Federal Ministry for Economic Cooperation and Development (BMZ). GIZ has introduced the hospital partnership programme in 2016, with the goal to pair German hospitals with hospitals in LMIC for knowledge exchange, mutual learning and common training programmes to eventually enhance the quality of care. This programme is not restricted to certain diseases or disciplines.

During the 1st International Conference on Hospital Partnerships in Berlin in October 2022, a workshop on Advancing Cancer Care has focused on the key issues in cancer care in LMIC and discussed challenges and possible solutions in-depths for 2 days. We would like to draw attention to the results of this workshop as it gives a unique insight gained from multi-national discussion among cancer specialist from seven LMIC and their German cooperation partners.

Five topics have been identified to play a key role in the effort to reduce the cancer burden in LMIC: *Health Policy & Financing*, *Barriers to Access*, *Capacity Building*, *Cancer Registries* and *Adapted Treatment Guidelines*. Figure 1 displays the interconnection between the five core topics (in blue), and related main topics (in red) and other important implicated factors (in green). Furthermore, stakeholders (in yellow) in the field of cancer service delivery have been identified as well. This mind map—as the result of the multi-national in-depths discussions—illustrates the different societal fields that need to be addressed to mitigate the various existing challenges and ultimately to reduce cancer diagnosis in late stages.

**Fig. 1:**
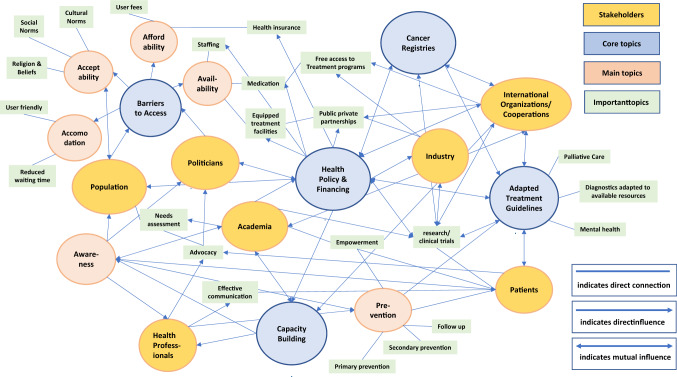
Mind-mapping of the complex field of cancer care in LMIC as discussed by the multi-national participants of the GIZ workshop *advancing cancer care*. (Color figure online)

Hereby, international organizations & cooperations can be instrumental in earlier management of cancer when focusing on capacity building, development of adapted treatment guidelines and cancer registries but also in consulting health policy strategies. These mentioned fields carry the opportunity for mutual learning and development. Additional important areas of cooperation/consultation for international partnerships are public private partnerships, free *access to treatment programs* and exchange of knowledge and expertise in the academic sector. Existing examples of these international engagements are given by the *NCCN harmonized guidelines™* for sub-Saharan Africa, various bi- or multi-national research cooperations, access to medicine programs like *The Max Foundation* and the *GIZ hospital partnership program*.

The broad cultural, social and religious dimensions that influence *barriers to access* of cancer services need to be addressed primarily by the LMICs themselves. International cooperations, however, can be conducive in promoting the acceptability of modern cancer services (from prevention to treatment and palliative care) through supporting cancer awareness campaigns.

To leverage the *political will* of the respective governments for action on non-communicable diseases as a whole and particularly on cancer control has been expressed throughout the discussions as essential. An essential contribution will be to assist policy makers in formulating and endorsing affordable and effective health policies. This includes also creating political frameworks for the private sector and the pharmaceutical and medical industries. Political commitment was also formulated by the European Society of Medical Oncology previously [5] as a way forward to fight the global cancer burden [5].

Another lesson learned from this workshop is the similarity of challenges among the participating representatives from different LMIC.

In conclusion, the authors of this letter emphasize on the importance of building international long-term cooperations, exchange programs and mutual learning experiences to advance oncology care on a global scale.

## Data Availability

Does not apply for this manuscript.
